# Synthesizing a Calcium Lignosulfonate Composite Water Retention Agent and Evaluating Its Regulatory Effect on Water Evaporation and Crack Evolution in Saline–Alkali Soil

**DOI:** 10.3390/gels12080734

**Published:** 2026-08-17

**Authors:** Xiaojing Chen, Baichuan Li, Zhiping Yang, Ke Wang, Xiaodi Guo, Hua Li

**Affiliations:** 1 Institute of Loess Plateau, Shanxi University, Taiyuan 030006, China; chen-xiao-jing1985@sxau.edu.cn; 2 Soil Health Laboratory in Shanxi Province, Institute of Eco-Environment and Industrial Technology, Shanxi Agricultural University, Taiyuan 030031, China

**Keywords:** hydrogel, water retention, calcium lignosulfonate, evaporation, crack, drying–wetting cycles

## Abstract

In this study, we synthesized a lignin-based superabsorbent hydrogel (LWR) to relieve severe evaporation and structural degradation in inland saline–alkali soils. The LWR was prepared via free-radical graft copolymerization of calcium lignosulfonate (CL) and acrylic acid (AA), with its swelling performance optimized systematically. Fourier transform infrared spectroscopy (FTIR) and scanning electron microscopy (SEM) were used to characterize its chemical and microscopic structure, and the soil column was exposed to three drying–wetting cycles to explore the effects of hydrogel dosage on soil evaporation and crack evolution. CL may graft into polyacrylic acid under optimal conditions (60% AA neutralization, 4% CL, 1% initiator, and 0.03% crosslinker) to form a porous hydrophilic 3D network. Consequently, the optimized LWR achieved swelling capacities of 1480 g/g and 122 g/g in deionized water and a 0.9% NaCl solution, respectively, showing high water absorbency and salt resistance. During cyclic drying and wetting, soil evaporation was first dominated by the hydrogel’s water retention capabilities; then, it was controlled physically by soil surface cracks. A moderate LWR dosage of 0.3% was used to maintain stable water retention in soil and the intact soil structure, which likely occurred due to its strong water absorption and hypothesized calcium ion bridging anti-cracking interactions. This work overturns the traditional view that a higher hydrogel dosage yields better water retention. Instead, it highlights the importance of conducting a long-term joint evaluation of the hydrogel’s water retention capacity and its resistance to soil dry–wet deformation stress, thereby offering theoretical support for eco-friendly water retention agent design and saline–alkali land remediation.

## 1. Introduction

Soil salinization is one of the major stress factors restricting global agricultural production and sustainable ecological development [1,2]. Statistics show that the area of saline–alkali soil worldwide exceeds 800 million hectares [3] and is expanding continuously due to climate change, irrational irrigation, and other human activities [4]. High concentrations of soluble salts, especially sodium ions, in saline–alkali soil raise its osmotic potential, degrade its physical structure, and decrease nutrient availability, thereby severely inhibiting crop growth [5,6]. Among various governance methods, chemical improvement and water management are the core links [7]. Specifically, salt accumulation in inland saline soil can be reduced by decreasing evaporation, while inhibiting capillary rise can effectively prevent saline groundwater from reaching the soil surface in coastal saline soils [8]. A water retention agent is a type of functional material with high water absorption capacity. It can absorb and slowly release water, improve soil water retention capacity, regulate the water–salt balance in soil, and optimize the soil structure, thus playing a positive role in improving saline–alkali land [9,10].

Conventional water retention agents comprise polyacrylamide (PAM)- and polyacrylic acid (PAA)-based polymeric hydrogels. Additionally, owing to their three-dimensional hydrophilic crosslinked networks, such materials possess an extremely high swelling performance and are capable of absorbing hundreds to thousands of times their own weight in deionized water [11,12,13]. However, their water absorbency decreases significantly when cations are present in the solution [14]. Incidentally, saline–alkali soil contains abundant soluble cations, especially sodium ions. Moreover, carboxylate ions (−COO^−^) on the molecular chains of polymeric hydrogels are subject to the ion shielding effect, which causes a significant decline in water absorption capacity. In addition, high salt concentrations reduce the osmotic pressure difference inside the polymer network, further shrinking the water absorption space [15]; this limitation causes the water absorption capacity of many conventional polymeric hydrogels to drop sharply when applied in saline–alkali land, greatly shortening their effective service life and even turning them into potential microplastic pollutants in soil [16]. Therefore, improving the water absorption performance of conventional polymeric hydrogels under salt stress has become a key issue that urgently needs to be solved in this field [17].

In recent years, research on synthesizing composite hydrogels using natural renewable biomass resources has attracted widespread attention [18]. As the second most abundant natural polymer in nature, lignin contains various reactive functional groups, such as complex phenolic hydroxyl, alcoholic hydroxyl, carboxyl, and sulfonic acid groups, and features favorable water absorption capacity and biodegradability [19]. Recent advances have demonstrated the potential of lignin-based hydrogels in addressing salt resistance challenges. For instance, lignin-modified PAM hydrogels and fenton-oxidized lignin-based hydrogels have been shown to improve the salinity resistance of polymer networks via hydrogen bonding and molecular crosslinking, alleviating salt-induced performance degradation. Additionally, lignin-modified composite hydrogels exhibit improved mechanical properties [20,21,22]. Calcium lignosulfonate is a major by-product of the sulfite pulping process. The sulfonic acid groups introduced into its molecules enhance water solubility, while the presence of calcium ions endows it with ion exchange and cementing properties [23]. Thus, introducing calcium lignosulfonate into the polyacrylic acid network system can not only partially replace petroleum-based monomers but also reduce material costs. More importantly, lignin’s polyphenolic structure allows it to undergo complexation or ion exchange with cations in salts, thereby alleviating the ion shielding effect [24]. Meanwhile, existing studies indicate that lignin is capable of promoting the formation and stability of soil aggregates via cementation effects under soil conditions, which may synergistically suppress crack development in saline–alkali soil during cyclic drying and wetting events [25,26]. Although existing studies have revealed lignin’s potential to enhance the salt resistance of hydrogels, further research is still required on the synthesis optimization of calcium lignosulfonate to improve the salt resistance of polyacrylic acid hydrogels. Furthermore, current research mainly focuses on the properties and structures of hydrogel materials themselves while neglecting their regulatory effects on soil during drying–wetting cycles, particularly soil evaporation and crack development, which are critical factors for the improvement of saline–alkali land.

This study aims to break through the salt resistance bottleneck of conventional polymeric hydrogels and proposes a novel multi-functional and eco-friendly composite hydrogel. Specifically, acrylic acid (AA) and calcium lignosulfonate (CL) were used as the polymerization monomer and the functional skeleton material, respectively. Additionally, a simple and efficient ultrasound-assisted aqueous solution polymerization method was adopted in this work to prepare a lignin-based composite hydrogel (LWR). The ratios of the preparation components were systematically optimized based on their swelling performance in deionized water and a 0.9% NaCl solution, which were used as evaluation indicators. Moreover, cyclic drying–wetting simulation experiments were conducted in soil columns containing saline–alkali soil to compare the LWR under the optimal ratio against a commercially available polymeric hydrogel (PWR). Unlike most existing studies that only focus on material characteristics, this study innovatively focuses on investigating the differences in the regulation effects of hydrogels on soil evaporation and crack development during repeated drying–wetting cycles, which are key links to the improvement in saline–alkali land. It also aims to determine the optimal application rate of the LWR and its dual function of water retention and crack resistance in saline–alkali soil. The results of this study will provide new ideas for material design and a theoretical basis for developing multi-functional and eco-friendly water retention agents suitable for improving saline–alkali land. Additionally, it will fill the research gap on the role of lignin-based hydrogels in regulating saline–alkali soil evolution under cyclic drying–wetting.

## 2. Results and Discussion

### 2.1. Optimizing the Water Absorption Performance of the LWR

#### 2.1.1. Effect of Neutralization Degree of Acrylic Acid (AA)

**Figure 1:**
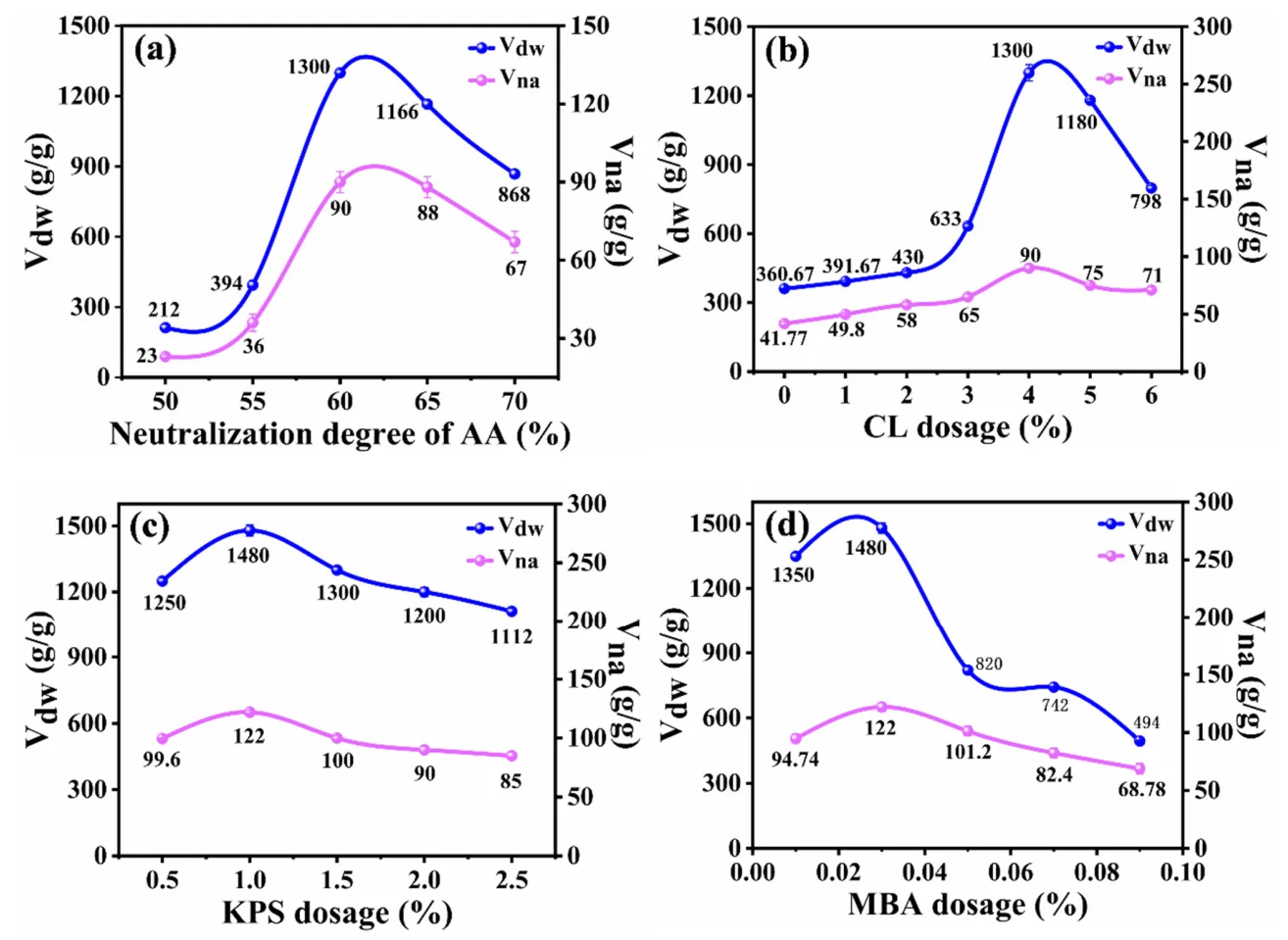
Effect of the synthesis parameters on the LWR’s swelling capacity in deionized water (V_dw_) and a 0.9% NaCl solution (V_na_): (**a**) acrylic acid (AA) neutralization degree; (**b**) calcium lignosulfonate (CL) dosage; (**c**) potassium persulfate (KPS) dosage; (**d**) N,N′-methylenebisacrylamide (MBA) dosage. Data are presented as the mean ± standard deviation (SD) (*n* = 3).

Figure 1a shows the effect of AA neutralization degree on water absorption performance. Swelling capacity in deionized water (V_dw_) and a 0.9% NaCl solution (V_na_) rose and subsequently declined with increasing neutralization, peaking at 60% neutralization with V_dw_ = 1300 g/g and V_na_ = 90 g/g. Specifically, moderate neutralization enriches −COO^−^ to boost interchain electrostatic repulsion and hydration for improved water uptake, while excess neutralization introduces abundant free K^+^ that generates ion shielding, suppressing network stretching and lowering absorbency [27,28].

#### 2.1.2. Effect of Calcium Lignosulfonate (CL) Dosage

Within a 0–4% CL dosage range, V_dw_ increased from 360.67 to 1300 g/g, while V_na_ rose from 41.77 to 90 g/g (Figure 1b). Appropriate CL modifying supplies extra –SO_3_^−^ hydrophilic sites and rigid crosslink junctions to counteract the decline in osmotic pressure induced by salt ions, thus improving the hydrogel’s hydration capacity and salt resistance [29,30]. CL dosages exceeding 4% cause an overabundance of localized crosslinking from bulky lignin macromolecules, shrinking the network’s free volume and reducing the swelling capacity of pure water and saline [31].

#### 2.1.3. Effect of Initiator Potassium Persulfate (KPS) Dosage

Figure 1c shows that the KPS dosage exerted a significant influence on the properties of the LWR, which presented a trend of increasing first and then decreasing. Swelling capacities (V_dw_/V_na_) rose to peak values of 1480 g/g and 122 g/g at 1% KPS. Additionally, a 1% KPS dosage produced balanced radical concentrations, which allowed homogeneous polymer frameworks with ideal chain length for water permeation and retention to form. In contrast, insufficient KPS yielded limited radicals, incomplete polymerization, and defective networks, while excess KPS induced rapid, premature chain termination, disrupting network integrity and reducing absorbency [32,33].

#### 2.1.4. Effect of Crosslinker N,N′-Methylenebisacrylamide (MBA) Dosage

As shown in Figure 1d, swelling increased then declined with rising MBA loading, reaching maxima of 1480 g/g (deionized water) and 122 g/g (0.9% NaCl) at 0.03 wt% MBA. At 0.09 wt% MBA, the values dropped sharply to 494 g/g and 68.78 g/g. Thus, moderate MBA content resulted in uniform, flexible networks with ample swelling voids, while excess MBA raised the crosslink density excessively, tightening the polymer matrix, limiting chain extension, and hindering water ingress to lower swelling capacity [34,35].

### 2.2. Structure of the LWR

**Figure 2:**
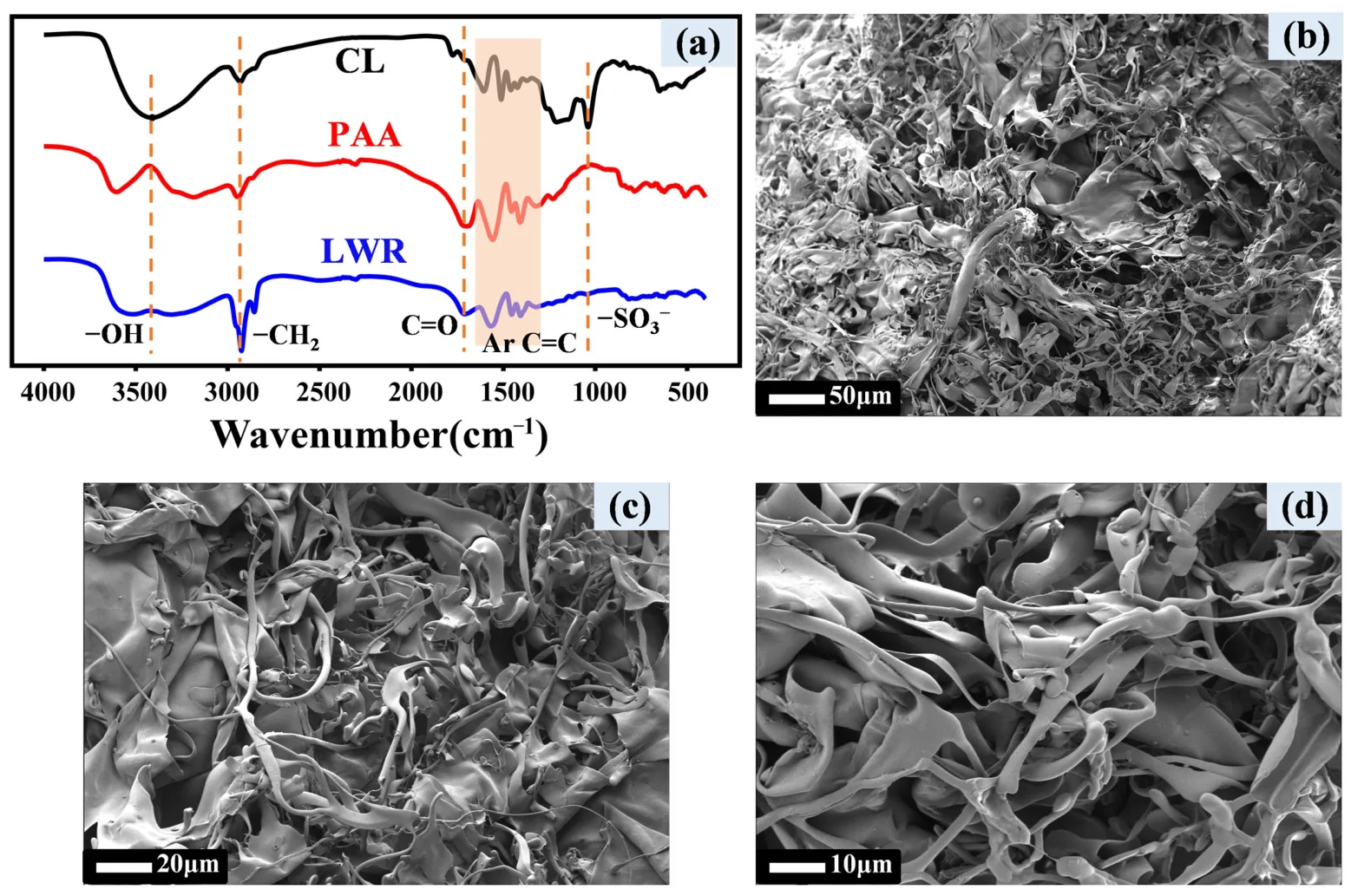
Characterization of the LWR: (**a**) FTIR; (**b**) 200× SEM image; (**c**) 500× SEM image; (**d**) 1000× SEM image. CL (pure calcium lignosulfonate), PAA (neat crosslinked polyacrylic acid).

The optimized LWR, neat crosslinked polyacrylic acid (PAA), and pure calcium lignosulfonate (CL) were characterized via FTIR (Figure 2a). Pure CL exhibits a broad −OH stretching band originating from phenolic and alcoholic hydroxyl groups on lignin skeletons at 3410 cm^−1^, as well as aliphatic C–H stretching at 2980 cm^−1^. Additionally, aromatic C=C skeletal vibrations characteristic of lignin appear at 1450–1605 cm^−1^, and the peak at 1040 cm^−1^ corresponds to symmetric stretching of sulfonate (−SO_3_^−^) groups [15,36]. Neat PAA shows dominant absorption at 1705 cm^−1^ (carboxylic C=O stretching) and 1555 cm^−1^ (ionized −COO^−^), with a single hydroxyl band from hydrogen-bonded carboxyls and no lignin-derived aromatic or aliphatic signals. The LWR’s spectrum is not a simple linear superposition of the two raw materials, thereby confirming potential chemical interactions and covalent grafting rather than physical blending. The −OH band at 3410 cm^−1^ broadens drastically after copolymerization due to the overlapping hydroxyl signals of grafted lignin and PAA carboxyl groups with altered intermolecular hydrogen bonding interactions. The aliphatic C–H peak at 2980 cm^−1^ is significantly intensified in the LWR, demonstrating that acrylic acid polymerizes to form abundant saturated −CH_2_ backbones covalently linked to lignin fragments. A distinct strong peak at 1705 cm^−1^ inherited from PAA verifies that massive hydrophilic carboxyl moieties are introduced into the composite network. Meanwhile, lignin’s aromatic C=C vibration signals (1450–1605 cm^−1^) are fully retained but broadened and enhanced by the newly generated −COO^−^ band at 1555 cm^−1^. The weakened intensity of the −SO_3_^−^ peak at 1040 cm^−1^ is potentially caused by spectral shielding from densely entangled PAA graft chains and strong absorbance of carboxylate side chains [18,32].

Collectively, the coexistence of characteristic peaks from both CL and PAA with obvious peak shape/intensity shifts confirms that calcium lignosulfonate macromolecules are covalently incorporated into the crosslinked polyacrylic acid matrix via free-radical grafting. Additionally, the integrated polar functional groups (−COO^−^, −SO_3_^−^, and −OH) within the hybrid three-dimensional network lay a structural foundation for the LWR’s high water absorbency and salt resistance.

The micromorphology of the optimized LWR is displayed in Figure 2b–d. The overall surface presents an intricate interwoven network structure constructed by abundant curly fibrous polymer chains and stacked wrinkled lamellar fragments derived from grafted calcium lignosulfonate segments. These dense, mutually entangled folds endow the material with a highly rugged surface morphology. Moreover, abundant irregular pores with diverse apertures are distributed throughout the intertwined network framework. These interconnected voids form unobstructed transport pathways running from the exterior surface into the inner matrix of the hydrogel. This hierarchical porous architecture may stem from successful graft copolymerization between CL and PAA. Rigid lignin macromolecular skeletons act as physical spacers that prevent excessive collapse and dense stacking of PAA molecular chains during polymerization and drying, consequently reserving sufficient open pore channels. Such structural characteristics bring two prominent advantages: Firstly, the interconnected pore networks provide unimpeded diffusion channels for rapid water infiltration and transport deep into the hydrogel interior, accelerating the water uptake rate. Secondly, the wrinkled rough surface carries abundant exposed hydrophilic functional groups (−COO^−^, −SO_3_^−^, and −OH), as verified using FTIR analysis, which can fully contact the aqueous solution and capture water molecules via hydrogen bonding and electrostatic interactions. Moreover, the robust interlocked network structure can effectively resist chain shrinkage induced by cation shielding in a saline environment, which lays a solid structural foundation for the favorable water absorption capacity and salt resistance of the LWR [20,37].

### 2.3. Effects of Hydrogels on Water Evaporation in Saline–Alkali Soil

**Figure 3:**
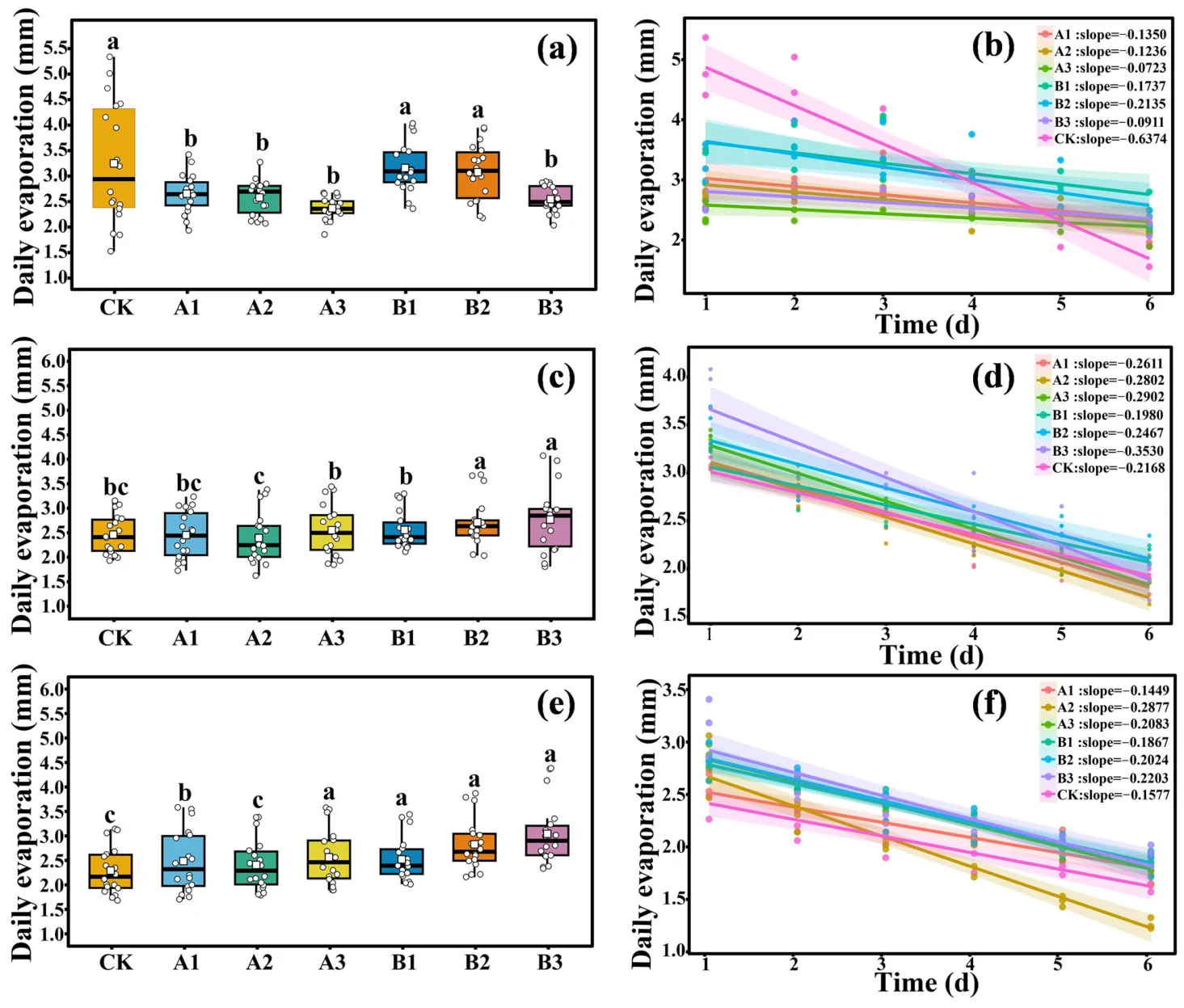
Variations in daily soil evaporation under three consecutive drying–wetting cycles: (**a**,**b**) Cycle 1, (**c**,**d**) Cycle 2, and (**e**,**f**) Cycle 3. CK (0% hydrogel), A1 (0.1% LWR), A2 (0.3% LWR), A3 (0.5% LWR), B1 (0.1% PWR), B2 (0.3% PWR), and B3 (0.5% PWR). In the boxplots (**a**,**c**,**e**), lines inside the boxes represent medians, square markers represent means, and error bars represent the standard deviation (S.D.) (*n* = 3). Different lowercase letters above the boxes indicate statistically significant differences in the main effect of treatments evaluated using two-way repeated measures ANOVA (*p* < 0.05) to account for non-independent repeated daily measurements from the same soil columns. The shaded region in (**b**,**d**,**f**) represents the 95% confidence intervals.

Daily evaporation rates of the soil across three drying–wetting cycles are summarized in Figure 3. Specifically, Figure 3a,b shows the variations in daily evaporation in Cycle 1. CK (control treatment without hydrogel) showed wider evaporation fluctuation and higher average evaporation than all hydrogel treatments. Group A (LWR) exhibited a narrower data dispersion than Group B (commercial PWR), while an elevated LWR dosage lowered the median evaporation and stabilized water release. Fitted evaporation slopes confirmed that CK had a steep loss rate (slope = −0.6374), while all hydrogel groups displayed a far milder decline (absolute slope: 0.00723–0.2135), with Group A consistently lower than Group B across the test period. This indicated that LWR had a better water retention effect than PWR. Specifically, in the intact initial soil structure, the LWR’s 3D hydrophilic network and hydrophilic groups (Figure 2) converted free water to bound water, flattening evaporation curves and cutting peak water loss [38].

Figure 3c,d show the variations in daily evaporation in Cycle 2. PWR (B1–B3) significantly raised the average evaporation relative to CK, and the promoting effect strengthened with dosage. High-dose LWR (A3) slightly increased evaporation; low-dose A1 showed no obvious difference from CK, whereas 0.3% LWR (A2) yielded the lowest evaporation. All Group B slopes exceeded CK, and high initial evaporation kept the B treatments above CK throughout the cycle. The absolute value of Group A’s slope also rose with the increase in application rate, exceeding that of CK. However, owing to the low initial evaporation, the daily evaporation of Group A gradually fell below that of CK as evaporation declined faster. This trend was most pronounced for A2, whose daily evaporation was significantly inferior to that of CK from the third day. This demonstrated that the 0.3% LWR application rate can remarkably suppress soil water evaporation in the middle and later stages. The results may be attributed to the damaged soil structure after Cycle 1, which gradually generated cracks. Such cracks acted as a critical factor affecting soil water evaporation. Lastly, 0.3% LWR achieved optimal water retention [39].

Figure 3e,f show the variations in daily evaporation in Cycle 3. PWR and high-dose LWR (A3) further accelerated evaporative loss compared with CK; only A2 maintained a lower mean evaporation (3.47% reduction vs. CK). The B series and A3 shared high initial evaporation and fast attenuation, but their daily loss remained higher than that of CK over the whole cycle. A1 showed similar evaporation trends to CK, with slightly higher values. Despite the minor difference in cumulative evaporation between A2 and CK in the third cycle, their evaporation behaviors differed markedly. A2 had the largest absolute slope value with drastic evaporation attenuation. Its initial daily evaporation value exceeded that of CK, yet its evaporative loss dropped rapidly over time, resulting in significantly lower daily evaporation than CK and other treatments in the middle and later stages. After two drying–wetting cycles, PWR and high-dose LWR treatments displayed similar evaporation behaviors, while low-dose LWR and CK also shared comparable evaporation performance. This further revealed that recurrent drying and wetting diminished the regulatory role of hydrogels in soil water evaporation. Additionally, the progressive deterioration of the soil structure likely became the core factor modulating evaporation [25]. The 0.3% LWR amendment may have effectively alleviated structural damage in soils and thus restrained water evaporation in saline–alkali soil [40].

### 2.4. Effects of Hydrogels on Crack Development in Saline–Alkali Soil

**Figure 4:**
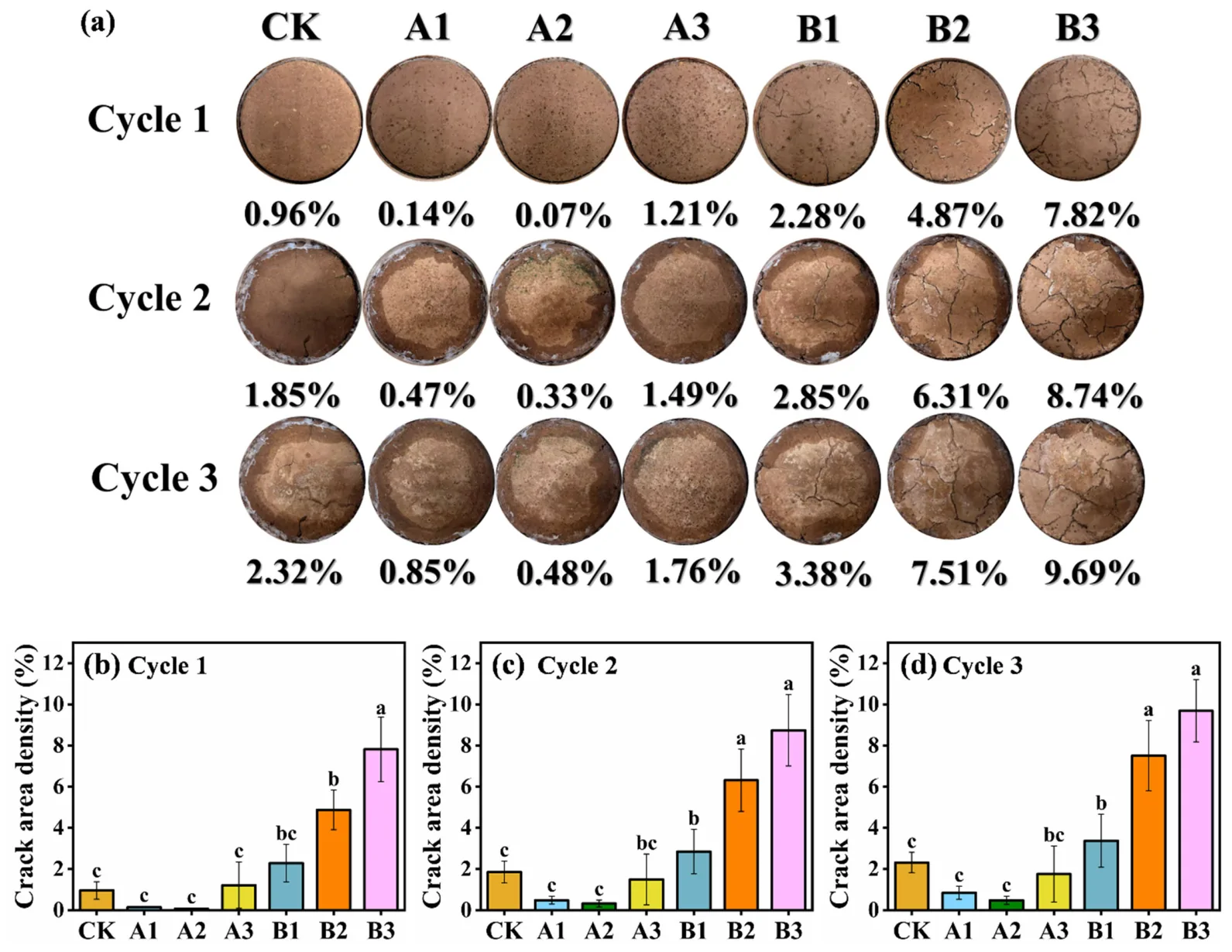
Dynamic evolution of surface cracking in saline–alkali soil across three consecutive drying–wetting cycles: (**a**) top-surface digital photos, (**b**) crack area density in Cycle 1, (**c**) crack area density in Cycle 2, and (**d**) crack area density in Cycle 3. CK (0% hydrogel), A1 (0.1% LWR), A2 (0.3% LWR), A3 (0.5% LWR), B1 (0.1% PWR), B2 (0.3% PWR), and B3 (0.5% PWR). Crack area density values are expressed as the mean ± standard deviation (SD) (*n* = 3). Different lowercase letters indicate significant differences at *p* < 0.05.

Crack evolution under cyclic drying–wetting is shown in Figure 4. Overall, the initiation and propagation of soil cracks were random. As the number of drying–wetting cycles increased, surface cracks continuously developed and expanded in all treatment groups. This revealed that cyclic swelling upon water uptake and shrinkage during dehydration caused cumulative structural damage to the soil. Additionally, soil stress was constantly released under multiple alternating drying and wetting processes, leading to a gradual increase in soil cracks with the rise in cycle numbers. The blank CK reached a crack area density of 2.32% after three cycles, reflecting the inherent cracking risk of saline–alkali soil under cyclic wetting and drying.

LWR (Group A) effectively restrained crack propagation, while A1 and A2 reduced crack density by 63.36–85.42% and 79.31–92.71%, respectively, relative to CK across all cycles. In contrast, high-dose A3 initially generated more cracks than CK but fell 24.14% below CK after Cycle 3. This anti-cracking performance is possibly due to calcium lignosulfonate’s hypothesized interparticle cementing effect. Excessive LWR aggravated volumetric fluctuation and weakened soil stabilization under alternating drying and wetting events [26]. Collectively, moderate LWR incorporation can better preserve the structural integrity of saline–alkali soils.

Conversely, commercial PWR (Group B) aggravated cracking in the order of B3 > B2 > B1 > CK. Even 0.1% PWR produced a crack density 1.46–2.38 times that of CK, and 0.5% PWR reached 4.18–8.15 times CK’s value. A plausible explanation for this observation is the weak interfacial bonding between PWR and the surrounding soil particles, which may have led to isolated hydrogel aggregates being distributed within the soil matrix. These aggregates swell upon hydration and shrink during dehydration. Thus, their dramatic volumetric fluctuations may generate intense localized stress, which represents a possible pathway driving the formation of crack initiation sites and fracture expansion across the soil matrix. The crack-promoting effect of PWR is amplified as its incorporation dosage rises [41]. Thus, the incorporation of a hydrogel substantially modulated soil cracking characteristics, with efficacy contingent upon its category and application dosage.

### 2.5. Correlation Between Water Evaporation and Crack Development

**Figure 5:**
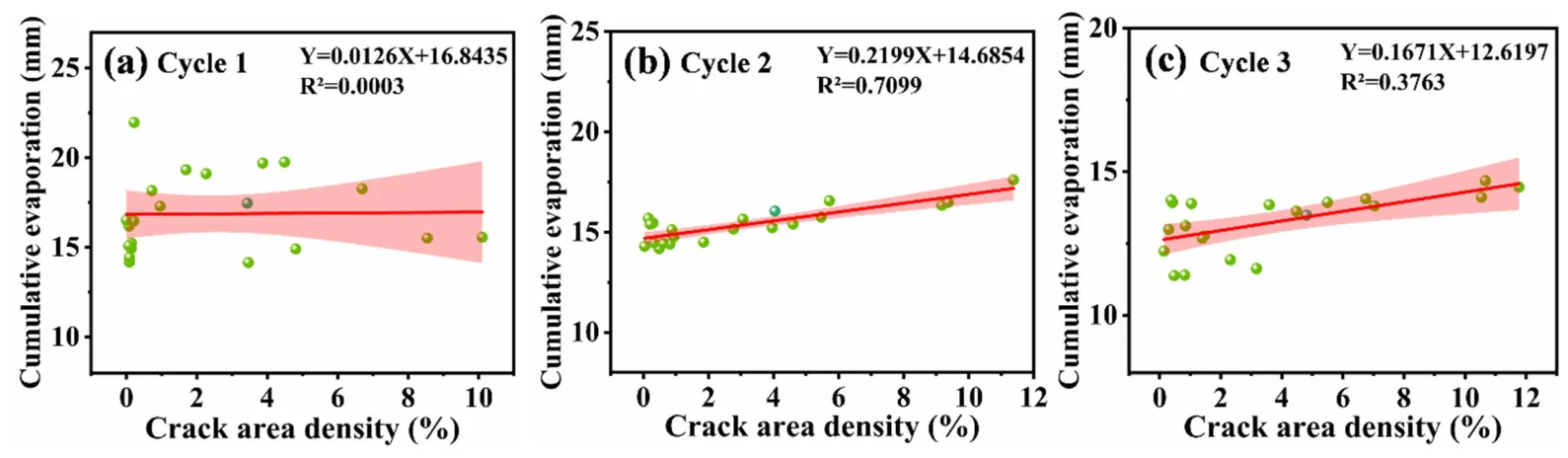
Correlation between soil crack area density and cumulative evaporation under drying–wetting cycles: (**a**) Cycle 1; (**b**) Cycle 2; (**c**) Cycle 3. The red solid lines denote linear fitted lines, and red shaded regions represent the 95% confidence intervals (*n* = 3, *p* < 0.05).

To further elucidate the regulatory mechanisms and patterns of LWR governing water evaporation and crack evolution in saline–alkali soils, linear regression between crack area density (X) and cumulative evaporation (Y) was conducted for each cycle (Figure 5). For Cycle 1 (Figure 5a), the fitted regression line was nearly horizontal with a slope close to zero, which was accompanied by a broad confidence interval (red shaded region). Data points were concentrated at a crack area density of approximately 2%, whereas the corresponding cumulative evaporation values scattered widely between 14.16 mm and 21.91 mm, yielding a coefficient of determination of R^2^ = 0.0003. This result reveals that crack density showed a negligible linear correlation with evaporation in Cycle 1. The intact soil matrix maintained continuous capillary channels, and water evaporation was dominated by capillary transport at this stage [25]. The hydrogels regulated water loss mainly via internal water immobilization rather than crack control, and LWR outperformed PWR in water retention due to its stronger water absorbency [15,17].

In Cycle 2 (Figure 5b), the fitted regression line exhibited an upward trend with a slope of 0.2199. The confidence interval narrowed substantially, and the coefficient of determination (R^2^) increased to 0.7099. These results demonstrate that variations in crack area density exerted significant linear control over soil evaporative loss, where cumulative evaporation rose synchronously with increasing crack area density. The calcium lignosulfonate fraction within LWR may reinforce interparticle bonding and limit crack expansion. In contrast, PWR aggregates may induce heterogeneous deformation and extensive macroscopic fractures [23,26]. Additionally, dense cracks increased the surface area for evaporation and became the dominant water-loss pathway [39,41]. Thus, the hydrogel type and dosage altered crack severity and final cumulative evaporation.

In Cycle 3 (Figure 5c), the coefficient of determination (R^2^) of the fitted regression line decreased to 0.3763 and was accompanied by a reduced slope of 0.1671. This result reveals that the dominant control of crack area density over soil evaporative-mediated water loss diminished substantially. Additionally, deep, continuous cracks prevented vertical capillary transport, causing rewetting water to be trapped in deep soil layers and preventing it from easily migrating upward [25,42]. This explains why the CK treatment exhibited low cumulative evaporation despite extensive cracking, corresponding to several outlying scattered data points in the figure. Meanwhile, Group B exhibited rising crack density but declining evaporation increments. For Group A, its well-preserved soil structure combined with the water retention capacity of the LWR facilitated moisture accumulation at the soil surface and promoted evaporation, thereby yielding higher evaporative amounts for A1 and A3 than CK. After three cycles, A2 only maintained a crack density of 0.48%, and its continuous soil matrix trapped water via the hydrogel’s superabsorbent networks to suppress surface evaporation [17,21].

### 2.6. Discussion

In this LWR system, calcium lignosulfonate may covalently graft onto PAA chains via free-radical copolymerization (Figure 2a). CL introduces two types of functional groups absent in single PAA: aromatic-ring-linked sulfonate (−SO_3_^−^) and ion-exchangeable Ca^2+^. Extra −SO_3_^−^ groups increase the total negative charge density of the network, compensating charge loss caused by Na^+^ shielding. Dissociated Ca^2+^ may act as ion bridges to crosslink −COO^−^ (PAA) and −SO_3_^−^ (CL), forming a hybrid ionic–covalent network skeleton. This hybrid structure is proposed to resist chain collapse under high salinity, delivering 90 g/g of saline absorbency compared to the 42 g/g of PAA (Figure 1b) [15,29]. A moderate neutralization degree, initiator, and crosslinking agent can form a uniform, stable, and complete three-dimensional network structure (Figure 2b–d), which provides abundant channels for rapid water infiltration and further enhances the water absorption performance of the LWR [31,32,33]. Additionally, it has better water absorption performance (V_dw_, V_na_) compared with most reported lignin/biomass-modified hydrogels, as shown in Table 1.

Commercial PWR only relies on a single water retention mechanism without soil structural modification, and its isolated swollen–shrunken aggregates generate local stress to trigger massive soil cracks (Figure 4). In contrast, Ca^2+^ released from the LWR during hydration creates hypothesized interparticle bridging between the negative sites in soil clay minerals and lignin fragments [21,26]. Ca^2+^ neutralizes the negative charges on the saline soil clay’s surface, reducing electrostatic repulsion between mineral grains and promoting microaggregate formation. The rigid aromatic backbones of CL embed into soil aggregates to enhance tensile strength against drying shrinkage [23,25]. The moderate dosage (0.3%) balances hydrogel volumetric fluctuation and the soil cementation effect, maintaining crack density below 0.5% across three cycles, which is far lower than that of CK and all PWR treatments (Figure 4).

The dominant evaporation control factor switches gradually with cumulative soil structural damage (Figure 5). In the initial stage with an intact soil structure, soil water evaporation is entirely controlled by the chemical water-holding capacity of the hydrogel. Specifically, unbroken capillary channels connect the topsoil to deep moisture. The LWR’s 3D network converts free soil water into non-volatile bound water via hydrogen bonding with the −COO^−^, −SO_3_^−^, and −OH groups, directly reducing daily evaporation peaks without crack interference [38,41]. In Cycle 2, the initial shrinkage creates surface fractures that multiply the effective evaporation area. The LWR’s calcium cementation restricts crack expansion, lowering the total evaporative surface and suppressing water loss. PWR cannot stabilize aggregates, so crack density and cumulative evaporation rise synchronously. The crack’s physical effect becomes the dominant factor governing water evaporation. In Cycle 3, deep cracks prevent vertical capillary transport, so most of the water is trapped in the subsoil and is unavailable for surface evaporation. Only 0.3% LWR simultaneously retains the continuous topsoil matrix and maintains long-term water-locking capacity, resulting in sustained evaporation reductions in the later stage of cyclic alternation. The soil’s water evaporation is co-regulated by multiple factors, including capillary interruption induced by structural cracking and the chemical water-locking effect of hydrogels [25,42].

**Figure 6:**
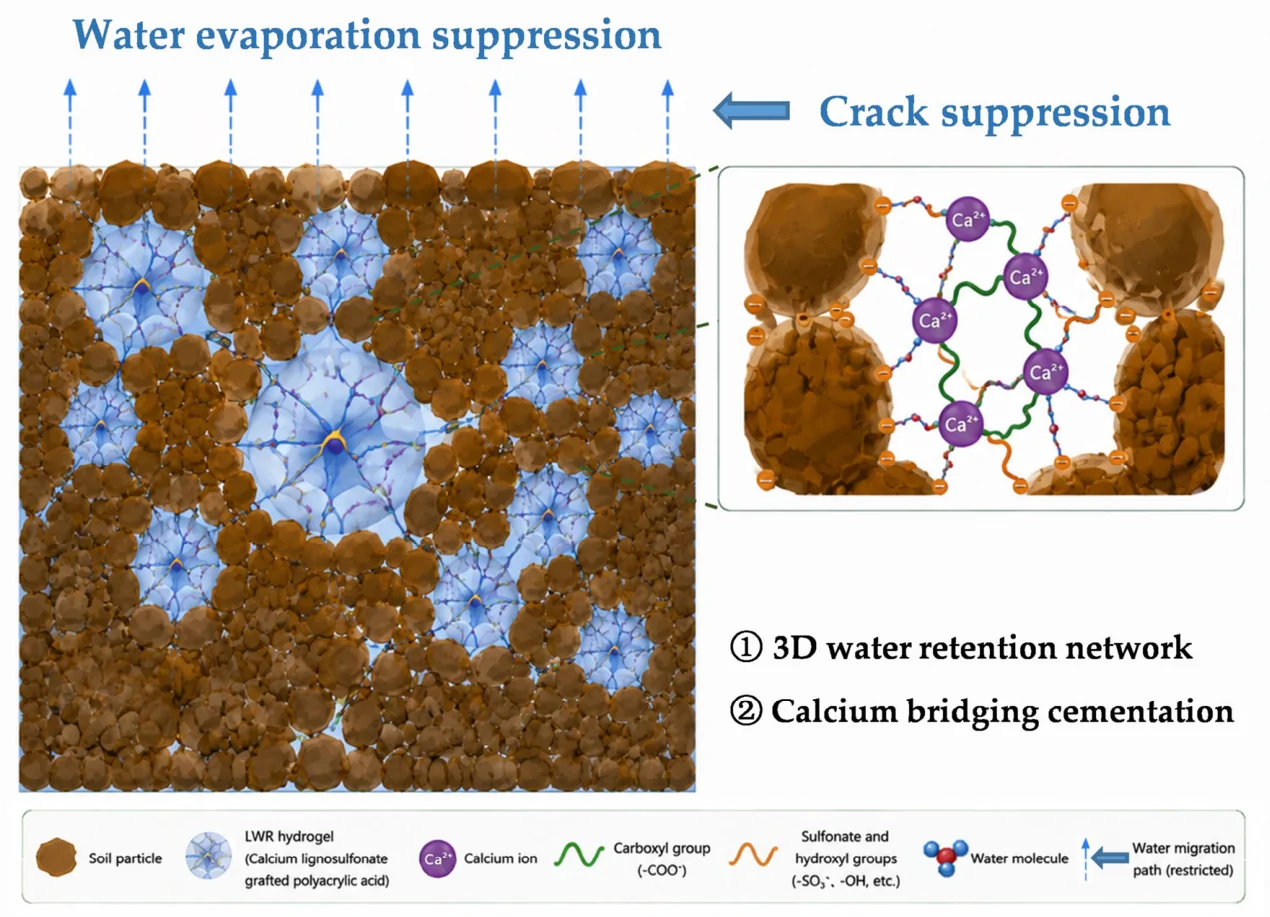
Schematic illustration of the saline–alkali soil improvement mechanism of the LWR.

In summary, the synergistic combination of a CL-derived salt-resistant hybrid network, calcium-mediated soil aggregate cementation, and its water-locking capacity constitutes the LWR’s dual-regulation mechanism of cycle-dependent evaporation for saline–alkali soil improvement. The schematic illustration of the mechanism is shown in Figure 6.

Unlike existing hydrogels, this study breaks through the salt resistance bottleneck and proposes a novel multi-functional and eco-friendly composite hydrogel (LWR). The LWR restrains soil surface cracks and evaporation, thereby realizing synchronous water conservation and soil improvement rather than water absorption alone. Previous studies focus on material characteristics and on evaluating its static water absorbency in a solution, which ignores cyclic environmental stress in actual farmland. This study focuses on the application effects of the LWR, which provides novel insight into dynamic water regulation under drying–wetting cycles in saline–alkali soil. Furthermore, it identifies the optimal application dosage as 0.3% and confirms that excessive application induces additional soil stress due to the uneven swelling and shrinkage of the gel volume and exacerbates cracking. This overturns the conventional belief that a higher dosage yields better performance and provides a quantitative basis for precise field application. This work provides innovative material design strategies and a theoretical basis for the development and engineering application of eco-friendly multi-functional water retention agents for saline–alkali soil improvement.

### 2.7. Limitations and Prospects

Despite the promising findings obtained in this study, multiple aspects require further exploration to comprehensively assess the practical applicability of the LWR in large-scale agricultural engineering.

#### 2.7.1. Environmental Safety and Biodegradability

Unlike a fully petroleum-based commercial polyacrylic acid hydrogel (PWR), which is difficult to degrade and risks microplastic accumulation in soil, the LWR uses the industrial by-product calcium lignosulfonate as the main functional raw material. Based on the existing literature, the lignin backbone can presumably be gradually decomposed by soil lignin-degrading fungi and actinomycetes into small-molecular phenolic substances within 1–3 years under natural soil conditions, which is expected to lower the risk of persistent plastic residue pollution. The grafted polyacrylic acid segment combined with lignin is reported to possess a higher biodegradation potential than the pure PAA hydrogel [19]. Meanwhile, the calcium ions released during degradation may supplement soil calcium supply and alleviate soil sodification [23], implying favorable environmental safety when applied to saline–alkali farmland. Nevertheless, systematic experiments on the biodegradability and biosafety of LWR remain to be carried out in future research.

#### 2.7.2. Long-Term Field Performance and Durability

The laboratory simulation in this work only set three drying–wetting cycles, which cannot reproduce multi-year seasonal alternation, rainfall leaching, and crop root interference in actual field saline–alkali soil. Thus, long-term field monitoring is needed to clarify the service life of the LWR under continuous cropping conditions, as well as its lasting regulation effect on soil evaporation and crack development after multiple years of drying–wetting alternation. In addition, field trials are needed to quantify the interaction between the LWR and salt leaching under natural rainfall to evaluate its long-term water–salt regulation stability.

#### 2.7.3. Economic Feasibility

Traditional superabsorbent polymers rely heavily on expensive, oil-derived monomers such as acrylic acid and acrylamide. By incorporating up to 4% calcium lignosulfonate—a low-cost industrial waste by-product—onto the synthetic polymer chain, the LWR may reduce the raw material cost per unit weight while maintaining high swelling ratios (1480 g/g in H_2_O and 122 g/g in 0.9% NaCl). Additionally, a moderate application dosage (0.3%) of the LWR can result in simultaneous water retention and anti-cracking effects, which may enhance the improvement effect compared with single-function commercial water retention agents. Accordingly, LWR exhibits potential economic advantages. Additionally, further research on the low-cost large-scale preparation of the LWR would be more economically feasible and broaden its application potential in saline–alkali soil improvement.

#### 2.7.4. Eco-Biological and Agronomic Responses

The present evaluations primarily centered on physical and hydraulic indices (e.g., evaporation dynamics, crack evolution characteristics, and water retention behavior). Therefore, systematic measurements of soil pH, electrical conductivity (EC), exchangeable sodium, particle size distribution and soluble ion composition were not completed within this study. Subsequent work must systematically investigate eco-biological and agronomic responses, such as soil pH, EC, exchangeable sodium, soluble ion composition, soil aggregate stability, nutrient retention, rhizospheric microbial community structure, crop growth status, root development and biomass accumulation to support its comprehensive application as a soil quality improvement amendment.

In addition, this study aims to synthesize an integrated lignin–polyacrylic acid composite hydrogel (LWR) and systematically characterize its overall comprehensive performance (salt resistance, water absorption, evaporation regulation, and crack resistance) under drying–wetting cycles in saline–alkali soil, thereby demonstrating the advantages of lignin composite modification in improving saline–alkali soil. Therefore, the insufficient mechanistic analysis constitutes an obvious limitation of this research. Future research will build upon comprehensive characterization analyses of the LWR’s structure and properties by designing single-component soil control experiments (soil + CL and soil + AA/PAA treatments) in conjunction with microscopic soil interface characterization, with the aim of fully elucidating the mechanisms of action, including calcium ion-mediated bridging and cementation effects, along with the underlying mechanisms of saline–alkali soil improvement. Meanwhile, further targeted experiments covering gradient soil types with varied salinity and alkalinity will be carried out in our follow-up research to systematically clarify the applicable threshold of LWR.

## 3. Conclusions

This work developed a calcium lignosulfonate-modified polyacrylic composite hydrogel (LWR) via ultrasound-assisted copolymerization, overcoming the poor salt resistance and soil cracking defects of conventional petroleum-based water retention agents. Under optimized synthesis parameters (60% AA neutralization, 4% CL, 1% KPS, and 0.03% MBA), LWR forms a porous 3D network with abundant sulfonate and carboxylate groups, achieving swelling values of 1480 g/g and 122 g/g in deionized water and a 0.9% NaCl solution, respectively, outperforming most reported lignin/biomass-modified hydrogels and commercial PWR.

A core novel discovery is the dosage-dependent dual regulatory effect under repeated drying–wetting cycles: a moderate 0.3% LWR application synchronously suppresses soil evaporation and crack propagation via hypothesized Ca^2+^ interparticle cementation, while a high LWR or full PWR dosage aggravates soil fracture and water loss. Additionally, correlation analysis further confirms that the dominant evaporation control factor shifts from hydrogel-driven chemical water locking to crack-driven physical water loss as soil structural damage accumulates across cycles, a mechanism rarely distinguished in prior hydrogel–soil studies.

Compared with single-function conventional superabsorbents, the LWR integrates low-cost biomass-based raw materials, potentially biodegradable skeletons, and soil structural protection. It breaks the conventional assumption that a higher amendment dosage delivers better water retention, thereby providing a targeted material strategy and theoretical support for large-scale eco-friendly saline–alkali land restoration.

## 4. Material and Methods

### 4.1. Materials

Acrylic acid (AA), calcium lignosulfonate (CL), N,N′-methylenebisacrylamide (MBA), potassium persulfate (KPS) as well as potassium hydroxide and sodium chloride were purchased from Macklin Biochemical Technology Co., Ltd., Shanghai, China. The commercially available polymeric hydrogel (PWR) is known as “Linzhibao” and was produced by Yida Agricultural Technology Co., Ltd., Handan, China. Its swelling capacity in distilled water and a 0.9% NaCl solution was 630 and 57 g/g, respectively. The saline–alkali soil was from Maozao, Shanxi Province, China, and was air-dried under natural conditions before being sieved through a 5 mm mesh. The sampling site is widely distributed, with moderate saline–alkali soil featuring obvious alkalization and salt accumulation phenomena. The soil type is castanozem.

### 4.2. Preparation of the LWR

We pipetted 20 mL of acrylic acid (AA, density = 1.05 g/mL, corresponding AA mass = 21.00 g) and performed partial neutralization by adding a 5 mol/L KOH aqueous solution dropwise at a rate of 1 mL/min under a 0–5 °C ice-water bath under continuous magnetic stirring at 300 rpm. After titration, the mixed solution was continuously stirred for 30 min to ensure a uniform pH distribution and complete the neutralization reaction. Different neutralization gradients (50%, 55%, 60%, 65%, and 70%) were accurately obtained by quantitatively adjusting the dosage of the KOH solution. The neutralization degree was defined as the molar percentage of AA neutralized by KOH and calculated using the following formula:

Neutralization degree (%) = (Moles of KOH)/(Total moles of AA) × 100%

**Figure 7:**
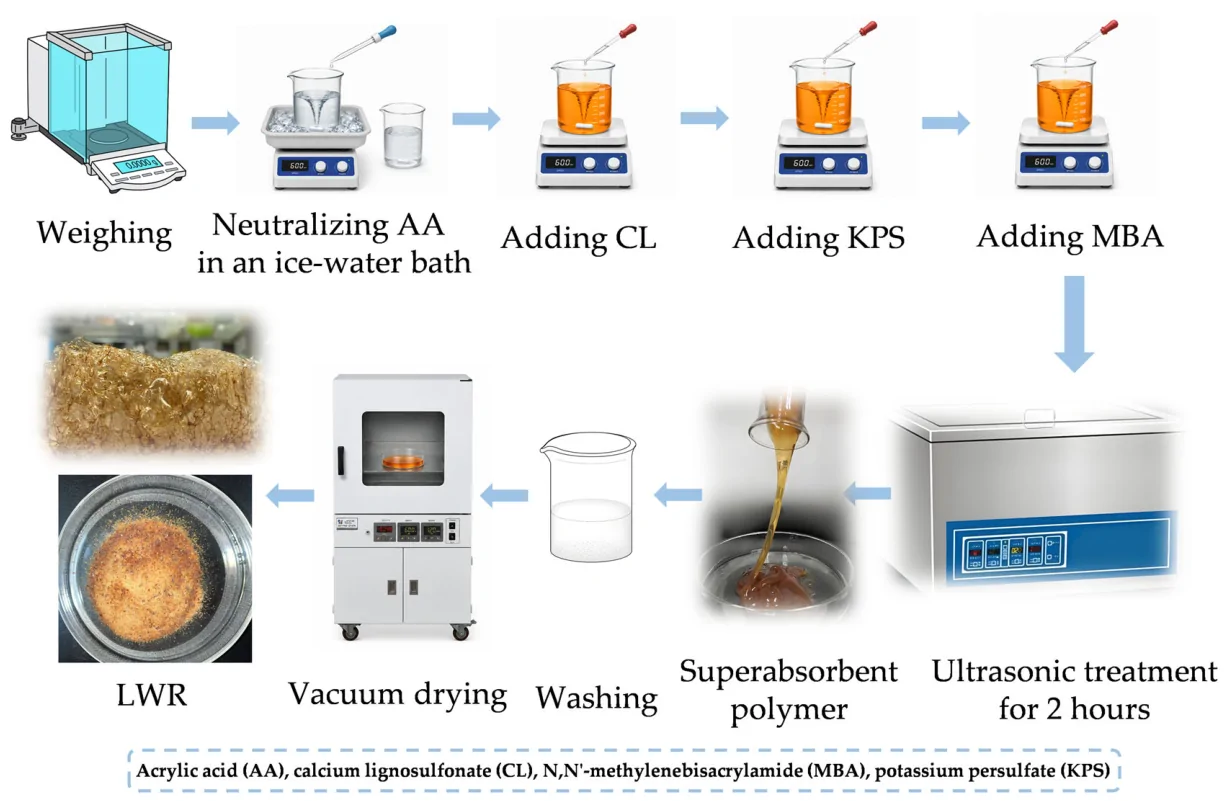
Schematic illustration of the synthesis procedures of the LWR.

**Figure 8:**
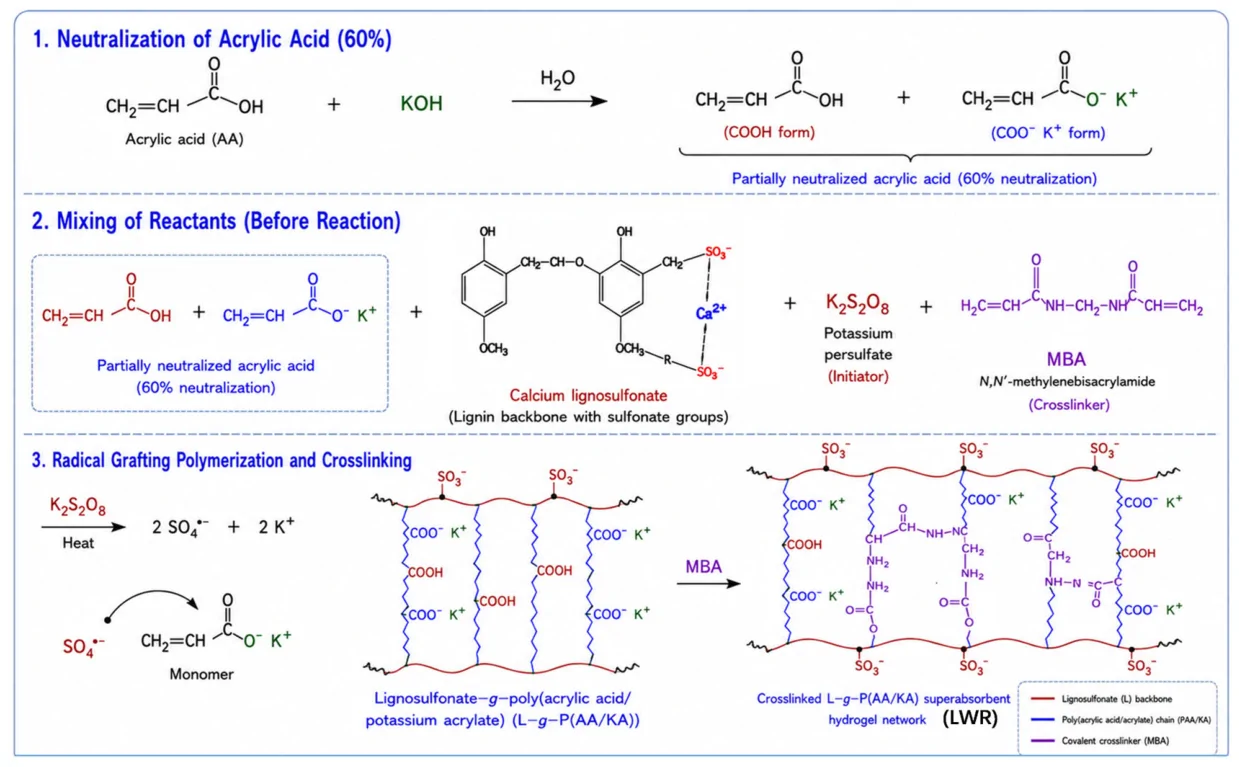
Schematic illustration of the synthesis mechanism of the LWR.

The neutralized AA solution was blended uniformly with a pre-dissolved calcium lignosulfonate (CL) aqueous solution. Next, we added the pre-dissolved initiator (KPS) and crosslinker (MBA). Then, CL, KPS, and MBA were added at designated dosages, with all percentage values of CL, KPS, and MBA based on the mass percentage relative to the absolute mass of the AA monomer (21.00 g). The mixed precursor suspension was continuously stirred for 5 min at room temperature; then, the beaker (250 mL capacity) was tightly sealed with plastic wrap and placed into an ultrasonic reactor for polymerization. Ultrasonic treatment was conducted under fixed conditions: a constant ultrasonic power of 510 W, a frequency of 40 kHz, and under continuous non-intermittent ultrasonic output mode. The temperature was raised from ambient (25 ± 1 °C) to 50 °C and maintained for 2 h to complete grafted copolymerization. After reaction termination, the product was washed 2–3 times with 200 mL of 20 vol% dilute ethanol per cycle, followed by 15 min of static soaking for each wash to extract unreacted AA monomers and soluble impurities and 3 additional rinsing cycles with 250 mL of deionized water per cycle for 30 min to remove residual inorganic salts. The purified product was transferred to glass Petri dishes and vacuum-dried at 110 °C under a vacuum pressure of −0.08 MPa until a constant mass was reached (mass variation < 0.001 g within 2 h). The dried solid block was then crushed and sieved through a 100-mesh standard screen to obtain a uniform LWR powder. Under the optimal synthesis formula (60% AA neutralization, 4% CL, 1%KPS, and 0.03% MBA), the average dry powder yield of the LWR reached 74.81% based on the raw material’s total solid input mass. The component ratios of CL, KPS, and MBA were systematically optimized based on their swelling performance in deionized water and the 0.9% NaCl solution, which were used as evaluation indicators. The preparation process and mechanism are depicted in Figure 7 and Figure 8, respectively.

### 4.3. Determination of Water Absorption Performance and Structure

The water absorption performance of the LWR was evaluated based on its swelling capacity in deionized water (V_dw_) and the 0.9% NaCl solution (V_na_, representing salt resistance). Additionally, it was tested in strict accordance with the agricultural industry standard NY/T 886-2022 [48] for agro-forestry superabsorbent polymers. Specifically, we placed 1.00 g of the LWR into 1000 mL of deionized water and 500 mL of the 0.9% NaCl solution, maintained at a constant temperature of 25 ± 0.5 °C. The solutions were then stirred for 5 min and left to stand for 30 min to allow the samples to fully absorb water and swell. The swollen gel aggregates were then transferred into a pre-weighed 80-mesh standard test sieve, and natural gravity filtration was conducted for 10 min. Next, the sieve was inclined, and residual free water was allowed to drain for another 10 min. We then recorded the total mass of the sieve plus the swollen gel (m_1_), that of the blank sieve (m_2_), and that of the initial dry LWR (m). The swelling capacity V (g/g) is calculated via the following formula:

$$V=\left(m_{1}-m_{2}\right)/m$$

**Figure 9:**
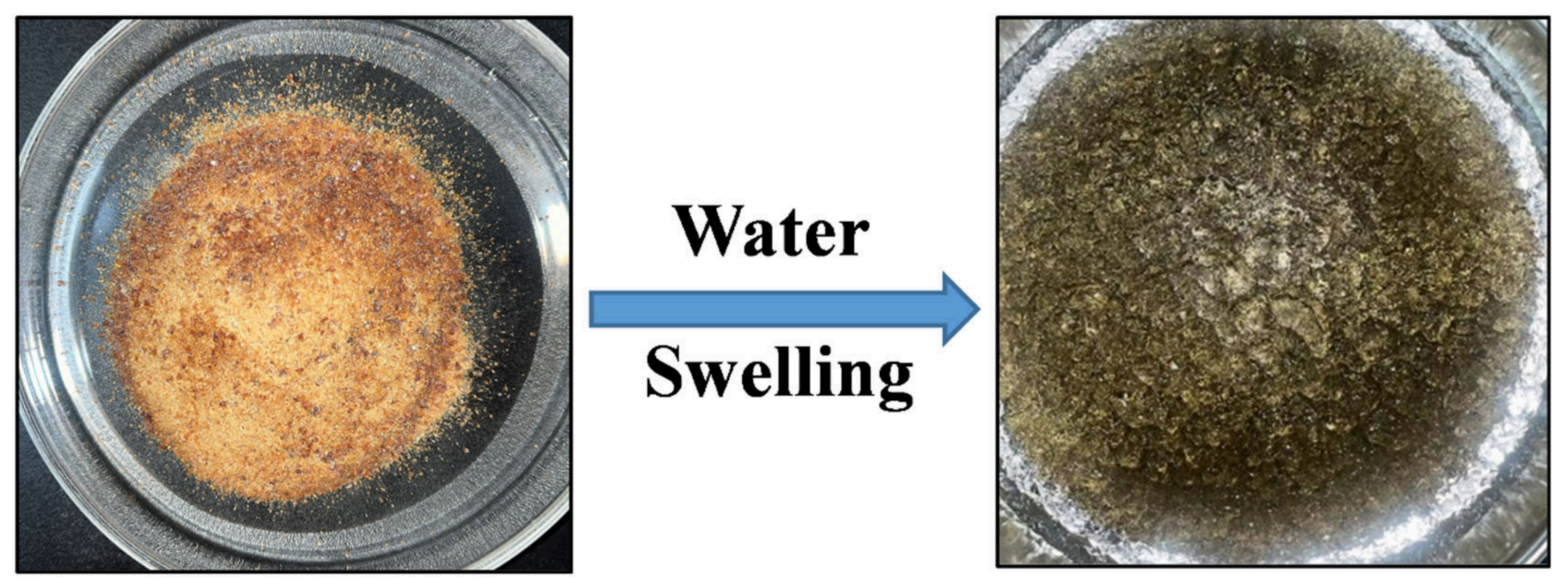
Image of the LWR before and after water swelling.

Three parallel replicates were measured for each group to calculate the average swelling value, and all swelling data were reported as the mean value ± standard deviation (SD). Digital photos of the dry powder and water-swollen LWR are shown in Figure 9.

The structure of the LWR was characterized using FTIR (Spectrum 3, PerkinElmer, Waltham, MA, USA) and SEM (Sigma 300, ZEISS, Oberkochen, Germany).

### 4.4. Drying–Wetting Cycle Experiments on Soil Columns

Each PVC column used in the experiment had a perforated bottom, a height of 20 cm and an inner diameter of 25 cm, and was uniformly coated with a thin layer of Vaseline on the inside to minimize sidewall flow. A total of 9.567 kg of air-dried saline–alkali soil was passed through a 5 mm sieve. The soil was then homogenously blended with 2 wt% desulfurized gypsum powder and back-filled into PVC columns under controlled compaction to maintain a fixed bulk density of 1.3 g/cm^3^. Then, the soil was irrigated to field capacity. Water at field capacity was completely retained via soil matric suction without free gravitational water; thus, no percolation or bottom drainage occurs despite having a perforated base. The perforated bottom only allowed ventilation and did not cause water loss. The 0–5 cm surface soil was taken out, air-dried, fully mixed with corresponding mass fractions of the LWR or commercial PWR hydrogel powder, and evenly refilled to restore the original 0–5 cm topsoil layer. All columns were irrigated a second time to the identical field capacity to unify the initial soil water content across all treatments. Seven experimental treatments were set up: CK (0% hydrogel), A1 (0.1% LWR), A2 (0.3% LWR), A3 (0.5% LWR), B1 (0.1% PWR), B2 (0.3% PWR), and B3 (0.5% PWR). Each treatment included 3 independent parallel soil column replicates, and all soil columns were placed in a controlled-environment chamber maintained at an ambient temperature of 25 °C and 30% relative humidity, with a fixed photoperiod of 12 h of light and 12 h of darkness per day. Rehydration irrigation was implemented once the volumetric water content of the soil in the blank CK group declined to 60% of the field capacity. Three complete drying–wetting cycles were carried out, with each single cycle lasting 6 days. In subsequent cycles, the irrigation amount was supplied uniformly based on the field capacity of the initial CK treatment. Since all irrigation events were controlled within the field capacity threshold, no gravitational drainage was generated throughout the experiment. During the experiment, soil columns were weighed at the same fixed time point (9:00 a.m.) every 24 h to calculate the daily water evaporation loss. Additionally, clear top-surface digital photos of each soil column were captured every 2 days for subsequent crack area quantification via Image J 1.54p software.

### 4.5. Data Analysis

Excel 2016 and SPSS 26 were used to analyze and calculate the experimental data, and Origin 8.5 was applied for plotting. Image J 1.54p was adopted to measure the area of soil cracks, and daily evaporation data based on repeated measurement data with temporal autocorrelation were obtained from continuous in situ monitoring of identical soil columns. A two-way repeated measures ANOVA (with treatment as the between-subject factor and time as the within-subject repeated factor) was performed to test the main effects and interaction effects, followed by post hoc tests for multiple comparisons (*p* < 0.05). All experimental groups were carried out in triplicate (*n* = 3).

## Figures and Tables

**Table 1:** Comparison of water absorption performance of different hydrogels in the literature.

Hydrogel	V_dw_ (g/g)	V_na_ (g/g)	Reference
SAP	544.95	44.0	[28]
SL-P(AA-co-VA)	949	62	[29]
St-g-AA-AM	1098	82.1	[32]
SW-AA-AM	738.12	90.18	[43]
Hydroxyethyl starch/calcium alginate-g-2-Acrylamido-2-methyl-1-propane sulfonic acid	1484	121	[15]
TSG-g-P [AA-co-AMPS/PVA]	1295	116	[44]
Starch-g-poly (AA-co-AM)/NCNPs	390	202	[45]
Poly (acrylic acid-co-acrylamide)/semicoke	643.13	79.82	[46]
WS/PAA–PAM	273.54	44.54	[47]
Calcium lignosulfonate composite hydrogel (LWR)	1480	122	This study

## Data Availability

The data presented in this study are available on request from the corresponding author.
